# Multistable internal resonance in electroelastic crystals with nonlinearly coupled modes

**DOI:** 10.1038/srep22897

**Published:** 2016-03-10

**Authors:** Christopher R. Kirkendall, Jae W. Kwon

**Affiliations:** 1 Micro-nano Devices and Systems Laboratory, Department of Electrical and Computer Engineering, University of Missouri, Columbia, Missouri 65211, USA

## Abstract

Nonlinear modal interactions have recently become the focus of intense research in micro- and nanoscale resonators for their use to improve oscillator performance and probe the frontiers of fundamental physics. However, our understanding of modal coupling is largely restricted to clamped-clamped beams, and lacking in systems with both geometric and material nonlinearities. Here we report multistable energy transfer between internally resonant modes of an electroelastic crystal plate and use a mixed analytical-numerical approach to provide new insight into these complex interactions. Our results reveal a rich bifurcation structure marked by nested regions of multistability. Even the simple case of two coupled modes generates a host of topologically distinct dynamics over the parameter space, ranging from the usual Duffing bistability to complex multistable behaviour and quasiperiodic motion.

The advent of micro- and nanotechnology revealed the inherently nonlinear dynamics that can manifest in resonant systems at even moderate forcing amplitudes. Consequently, there has been steady interest in exploiting nonlinear resonance in new ways. Notable in this regard is the use of bistability and hysteresis: in mechanical resonators to increase sensitivity and exceed the detection limit set by thermomechanical noise^1^,^2^,^3^,^4^, and in Josephson bifurcation amplifiers for high-speed readout of superconducting qubits^5^,^6^. Its presence in nanomechanical oscillators has enabled coherent signal amplification via stochastic resonance^7^ and the creation of bit storage and flip operations for controllable memory^8^,^9^. Most recently, thermo-mechanical relaxation oscillations were achieved by utilizing hysteresis in a quartz resonator^10^. However, these examples and much of existing literature employ single degree of freedom models that approximate an underlying continuous system^11^,^12^,^13^,^14^.

In this study we exploit nonlinear coupling between modes of an individual resonator driven by a single source to create regions of multistability in the frequency response beyond the usual bistability. Studies of coupling between individual resonators^15^,^16^ or arrays of them^17^,^18^ have introduced a host of nonlinear phenomena into the purview of micro- and nanoscale research, but fabrication, interfacing, and measurement of such systems offers many challenges. In some cases two driving forces are applied to a single resonator^19^,^20^. From an applications standpoint it would be easier if a single device forced by one source could generate the desired complex dynamics. Further innovation will benefit from a deeper understanding of nonlinear modal interactions in individual resonators modelled as multiple degree of freedom systems.

Recent experimental work has moved in this direction by exploring coupling between different eigenmodes of a single clamped-clamped beam^21^,^22^,^23^. Accounting for the effect of other modes enables precise determination of intra- and intermodal coupling coefficients. The ability to resolve the influence of various modes on the primary resonance is crucial as demands on nanoscale sensors increase. In many cases, however, the presence of the coupled mode does not change the bifurcation topology of the primary mode: the usual bistable Duffing behaviour persists. The coupled mode may shift or alter the shape of the primary mode Duffing response, but without changing the number of bifurcations or the existence of additional stable/unstable branches. Under proper conditions more complex interactions can arise that modify this basic structure, such as internal resonance. This phenomenon, well-studied in macroscale systems^24^,^25^,^26^,^27^, has received comparatively little attention at the microscale, despite its recent use to enhance frequency stability in a clamped-clamped silicon beam^28^. In part, this is likely due to the difficulty of designing devices that reliably exhibit internal resonances and other potentially useful complex dynamics – a difficulty compounded by a lack of systematic theoretical methods to quantitatively predict their occurrence. Thus, our understanding of modal interactions is far from complete, especially in systems where multiphysics couplings are prevalent (e.g. piezoelectric, thermoelastic).

Therefore, we derive a general model of nonlinear resonance in electroelastic crystals that accounts for both material and geometric nonlinearities and their effect on modal interactions. We use the model to explain heretofore unobserved manifestations of modal coupling in a quartz crystal resonator driven into the nonlinear regime. In addition to the well-known saddle-node bifurcations associated with Duffing bistability, we show that modal interactions can generate other saddle-node delimited, multistable regions that significantly complicate the dynamics. Our results indicate that even in the simplest case of two modes the nature of modal interactions can vary widely, and strongly depends on the system parameters: changes in the difference between eigenfrequencies of the associated linear system, the modal damping ratios, or nonlinear coupling coefficients can lead to topologically distinct bifurcation structures. This explains the aforementioned difficulty of ‘pinning down’ internal resonance and related effects in real devices. The framework presented here overcomes many problems of accurately predicting complex dynamics in electroelastic crystals.

## Theoretical Model

We now introduce the analytical-numerical approach to explain this behaviour. The analytical aspect consists in a perturbation analysis of the equations of electroelasticity. The result is a system of ordinary differential equations (ODEs) in terms of complex modal amplitudes (magnitude and phase of the response). The numerical aspect involves pseudo-arclength continuation^29^ of these modal equations to give the bifurcation structure in terms of excitation frequency and amplitude. The ability of continuation methods to quickly identify both stable and unstable solutions and their bifurcations, as well as continue periodic solutions arising from Hopf bifurcations, makes them instrumental to any study of modal interactions.

More importantly, the effect of a finite sweep rate can mask the underlying static bifurcation structure and prevent correct interpretation of both numerical integration and experimental results. As shown below, this is especially relevant at higher forcing amplitudes where thermal effects and bifurcation delay cause a number of transient behaviours. Numerical continuation allows one to separate these phenomena from the steady state dynamics, which are the primary focus of this paper. Lastly, even two coupled modes can generate multiple stable solutions that are experimentally inaccessible and difficult to locate numerically without detailed knowledge of their basins of attraction. Continuation methods resolve these difficulties, and once the bifurcation structure is known numerical integration can probe the dynamics of a given region of parameter space.

The equations of motion for nonlinear electroelasticity, in the absence of a body force, are defined by


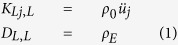


where *K*_*Lj*_ is the total first Piola-Kirchhoff stress tensor (material plus Maxwell electrostatic), *u*_*j*_ is the mechanical displacement and *D*_*L*_, *ρ*_*0*_ and *ρ*_*E*_ are the electric displacement, mass density and charge density, respectively, referred to the reference configuration (see Supplementary Information for exact definitions and details). In the restricted case of infinitesimal deformation these reduce to the familiar equations of piezoelectricity, and *K*_*Lj*_ becomes the Cauchy stress tensor. To account for a range of electroelastic interactions (piezoelectric, electrostrictive, etc.), constitutive relations for *K*_*Lj*_ and *D*_*L*_ are given in terms of a free energy function, *χ*. The exact functional dependence of *χ* can be tailored according to the level of generality desired^30^. Here we choose a simple polynomial dependence^31^ in terms of the Lagrange strain tensor *E*_*KL*_ and rotationally invariant electric variable *W*_*L*_* = φ*_*,L*_, where *φ* is the electric potential, such that *χ* = *χ* (*E*_*KL*_, *W*_*L*_). Since we are interested in the weakly nonlinear dynamics of electroelastic crystals, *χ* is expanded to cubic order in *u*_*j*_ and *φ* (see Supplementary Section 1)^32^.

The particular crystal symmetry and relevant electroelastic interactions determine further specification of the problem. The rotated Y-cut quartz used in experiments exhibits monoclinic symmetry and has negligible electrical conductance (*ρ*_*E*_ ≈ 0). Due to the relatively weak piezoelectric coupling of quartz, higher order electroelastic coupling terms are omitted from the constitutive relations^33^. In our experimental samples the longitudinal (thickness-stretch) and *x*_3_-polarized thickness-shear modes are far removed from the excitation frequency and not involved in any internal resonances with the *x*_1_-polarized thickness shear mode. Therefore, only the *u*_1_ component of displacement is considered, hereafter denoted as *u*. Thickness vibrations induced by a time-harmonic voltage applied to both faces of the plate are then governed by the following nondimensional equations and boundary conditions (see Supplementary Section 2):


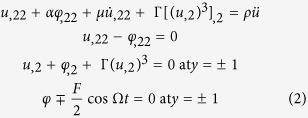


We proceed to reduce these to a set of amplitude modulation equations via the method of multiple scales^34^. Although equation (2) only has cubic nonlinearities, the general system given by equation (1) can possess quadratic terms as well. In this case, discretization methods can lead to qualitatively erroneous results^35^. Moreover, the presence of nonlinearities in the boundary conditions renders a Galerkin discretization inconvenient. For these reasons, the method of multiple scales is directly applied to the governing partial differential system to give equations of motion describing the time-evolution of coupled modes. One advantage of the multiple scales method is its ability to treat internal resonances between an arbitrary number of modes. Here we consider coupling between two thickness shear modes, designated by the indices *n* and *m*. The former is directly excited by a periodic signal (*Ω = ω*_*n*_* + εσ*_*1*_) and related to the latter by *ω*_*m*_ = 3*ω*_*n*_ + *εσ*_*2*_, where *ε* « 1 and *σ*_*1*_, *σ*_*2*_ are O(1) detuning parameters. The solution is sought in the form


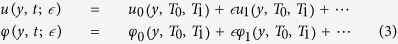


where *T*_0_* = t* is the fast timescale and *T*_1_ = *εt* is the slow time-scale on which the complex modal amplitudes vary.

The response, to first-order, is then given by


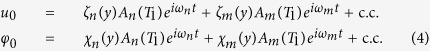


where *ζ*_*n*_(*y*), *ζ*_*m*_(*y*), *χ*_*n*_(*y*), and *χ*_*m*_(*y*) are spatial eigensolutions of the linear homogeneous system and c.c. denotes complex conjugation. The complex modal amplitudes *A*_*n*_ and *A*_*m*_ determine the bifurcation structure and are given by


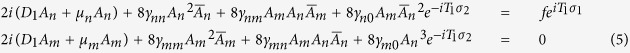


where *D*_1_ = *∂/∂T*_1_ and an overbar means complex conjugation. The undefined coefficients are determined by the eigensolutions of the linear system and its adjoint. Equation (5) is converted to a real-valued autonomous system of ODEs via the transformation


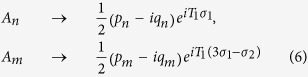


The slowly varying modal amplitudes and phases are then given, respectively, by *a*_*i*_ = 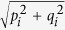 and *β*_*i*_ = arctan(*q*_*i*_/*p*_*i*_), where *i *=* n*,*m* (see Supplementary Section 3 for a full derivation).

## Results and Discussion

**Figure 1:**
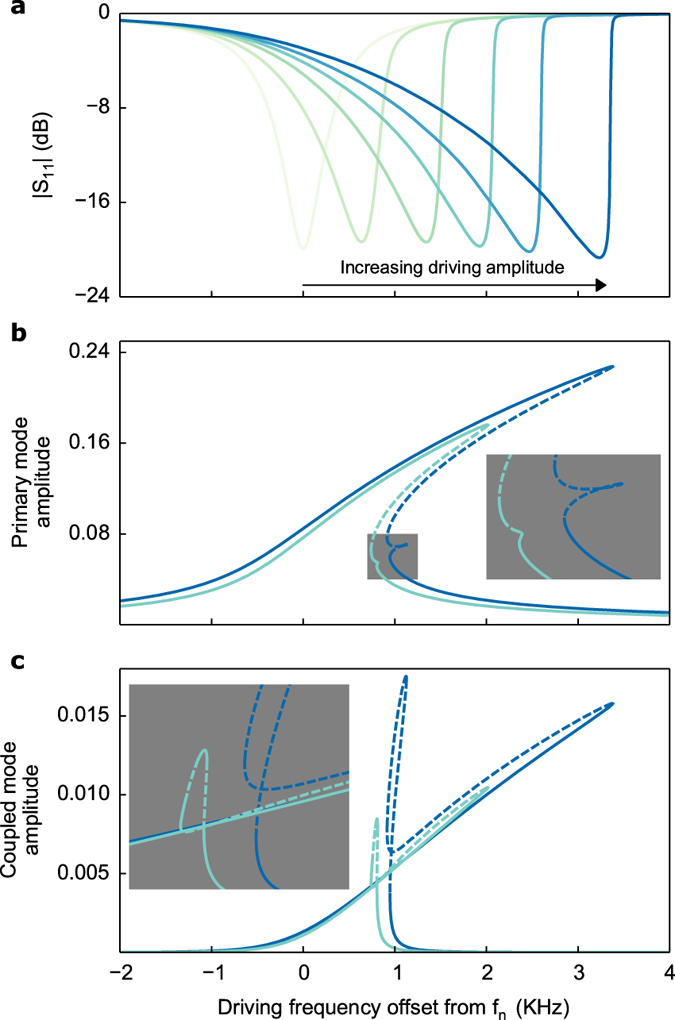
Duffing bistability and a precursor of multistability. (**a**) Experimental reflection parameter |S_11_| of the electroelastic crystal, showing progression from linear resonance to nonlinear Duffing bistability for a forward frequency sweep. Higher forcing amplitudes lead to greater bending in the response curve. (**b**) Theoretical response curve of the primary mode obtained by numerical continuation of equation (5). Solid (dashed) lines denote stable (unstable) equilibria. The curves correspond to two of the experimental curves in (**a**). The inset is a blow up of the offshoot from the lower branch that is a precursor of multistability at higher forcing amplitudes. (**c**) Same as (**b**) for the coupled mode amplitude.

We experimentally verify the model by measuring the signal reflected from the quartz resonator driven near its third thickness-shear harmonic (*f*_*n*_ = 30.01425 MHz). For driving amplitudes within the nonlinear regime yet below a certain threshold only the standard Duffing behaviour occurs, as shown in Fig. 1a. A decrease in the experimental reflection parameter |S_11_| corresponds to an increase in the theoretical modal amplitudes (compare Fig. 1a–c). However, even before the onset of more complex multistability the influence of modal coupling is apparent from the theoretical plots, in this case with the ninth harmonic at *f*_*m*_ = 90.045 MHz. As higher driving amplitudes cause more bending in the response of the primary mode (*a*_*n*_) the coupled mode amplitude (*a*_*m*_) grows in turn, though at this point *a*_*m*_ is practically negligible compared to *a*_*n*_ (Fig. 1b,c). Numerical continuation of equation (5) shows that the lower energy resonance branch begins to deform and eventually splits, generating the unstable loop in Fig. 1b,c. Note that the loop does not actually cross itself since each response curve—the primary mode *a*_*n*_ and the coupled mode *a*_*m*_—is a projection from four-dimensional space onto each mode (see Supplementary Section 4). Although the offshoot is unstable and thus not experimentally observable, this initial departure from simple Duffing behaviour anticipates the complex multistability witnessed at higher forcing amplitudes. Indeed, the small offshoot will later merge into a third stable branch, as detailed below.

It is important to contrast this behaviour with other kinds of modal interactions reported in the literature. In many cases the coupled mode has a trivial response until a critical driving amplitude, at which a pitchfork bifurcation appears in the upper branch of the bistable region^25^,^27^. After this point the coupled mode obtains a nontrivial response only over a certain bandwidth and is said to be internally resonant with the primary mode. The frequency response remains continuous after the pitchfork bifurcation appears, until finally jumping onto the lower branch after the saddle-node bifurcation.

**Figure 2:**
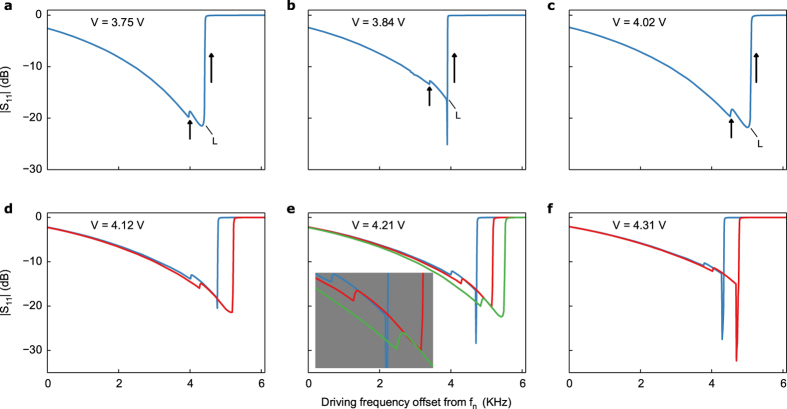
Existence of multistability. (**a–c**) Experimental reflection parameters for increasing drive voltages that demonstrate the existence of a third stable branch. The label L denotes saddle-node bifurcations. Transitions between stable branches are indicated by arrows in the direction of the transition. (**d**–**f**) Various sweep rates cause the system to transition to the third branch at different frequencies for the same drive voltage.

In contrast, for our electroelastic plate model the coupled mode always has a nontrivial response, even if it is practically negligible at lower forcing levels as in Fig. 1c. Moreover, unlike the former description of internal resonance, higher forcing amplitudes do not lead to pitchfork bifurcations. Rather, we witness the creation of additional pairs of saddle-node bifurcations and concomitant multistability in the frequency response. Instead of only two bifurcation points at least four such points exist, along with a region of three stable solutions nested within the previously bistable region. This is the most important prediction of the new model, which we experimentally demonstrate in Fig. 2 below. The majority of additional bifurcation points are not experimentally observable, however, as they exist on unstable portions of the solution (dashed lines in theoretical plots).

**Figure 3:**
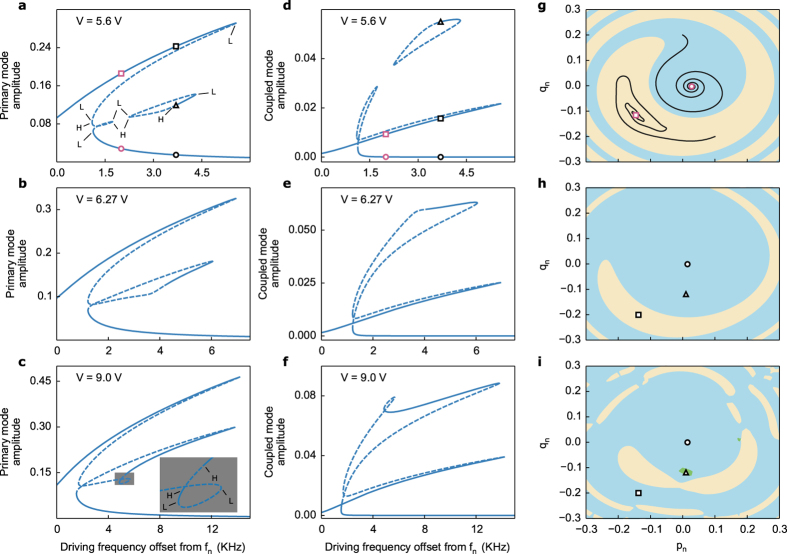
Passage from bistability to multistability. (**a**) Frequency response of the primary mode showing the existence of an isolated loop that contains a third stable branch. Labels L and H denote saddle-node and Hopf bifurcations, respectively. The circle and square magenta markers correspond to those in (**g**). The black markers correspond to those in (**h**) and (**i**). (**b**) At higher drive voltages the isolated branch merges with the offshoot from the lower branch. (**c**) Even higher drive voltages generate another saddle-node delimited, multistable region (blown up in the inset). (**d**–**f**) Same as (**a–c**) for the coupled mode. (**g**–**i**) Basins of attraction for the frequency response in (**a**,**d**). The black curves in (**g**) are trajectories leading to the two stable equilibria at 2 KHz offset. Panels (**h**,**i**) are at different initial conditions for the coupled mode.

During a frequency sweep, trajectories with an equilibrium on the higher energy resonance branch can switch to the new stable branch, as in Fig. 2. In terms of the theoretical plots the primary mode amplitude decreases (selecting the middle stable branch in Fig. 3a–c) and a portion of that energy is channelled to the coupled mode (selecting the upper stable branch in Fig. 3d–f). This corresponds to an increase in the observed reflection parameter |S_11_| since the resonator stores less vibrational energy. The transition is clearly evident in the reflected signals of Fig. 2. This has implications for potential technologies that exploit internal resonance for the feedback effect of the coupled mode on the driven mode, as demonstrated in ref. 28.

The passage from bistable to multistable behaviour is difficult to visualize from experimental data without theoretical foresight. When the frequency response is merely bistable, both branches can be reached by performing either a forward or backward frequency sweep. However, it is less straightforward when more than two stable solutions exist. Figure 3g–i shows the basins of attraction at two different frequencies for a number of equilibria that belong to the frequency response of Fig. 3a,d. Note that the plots do not represent the full basins of attraction since they are necessarily projected from the full space onto a single pair of initial conditions (*p*_*m*_(*t*_0_), *q*_*m*_(*t*_0_)) for the coupled mode. For equilibria in regions with only two stable solutions (Fig. 3g) the basins resemble those of a typical Duffing resonator. However, in regions with three stable solutions the basins strongly depend on the initial conditions of the coupled mode, as in Fig. 3h,i. In fact, the basin of attraction for an equilibrium point may lie entirely outside a plane of (*p*_*n*_(*t*_0_), *q*_*n*_(*t*_0_)) initial conditions defined by a given (*p*_*m*_(*t*_0_), *q*_*m*_(*t*_0_)), as occurs for the third stable equilibrium in Fig. 3h (triangle marker) and the higher energy one in Fig. 3i (square marker). A unidirectional frequency sweep may not be able to access these initial conditions, and thus a given measurement may not detect all stable solutions.

**Figure 4:**
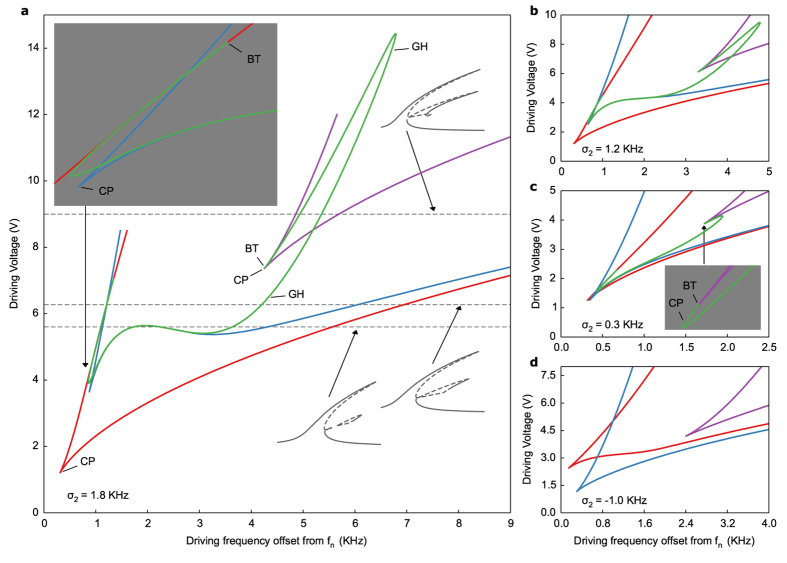
Bifurcation structure and codimension-two singularities. (**a**) Full bifurcation diagram in frequency-amplitude space. The red, blue, and purple curves are loci of saddle-node bifurcations and the green curve a locus of Hopf points. Labels CP, BT, and GH denote cusp, Bogdanov-Takens, and general Hopf (Bautin) bifurcations, respectively. The horizontal, dashed lines locate the frequency responses of Fig. 3a–f at their corresponding drive voltages in the full bifurcation diagram. (**b**–**d**) Same as (**a**) for different σ_2_ values.

The previous observations lend clarity to the experimental reflection parameters in Fig. 2. For example, the strong dependence of steady state amplitude on initial conditions translates into a similar dependence on the experimental sweep rate. While the main interest of this paper is the steady-state bifurcation structure of the system (namely multistability), we now explain why the choice of sweep rate can influence how the system selects a given stable solution. We take advantage of this dependence to elicit various responses that corroborate the theoretical plots in Figs 3 and 4. In Fig. 2d–f different portions of the third stable branch are mapped out by varying the sweep rate. In other words, the response transitions to the third branch at different frequencies in a way that depends on the sweep rate. A close inspection of the responses at a given forcing amplitude shows that they begin to slightly deviate from each other as the driving frequency increases, before each switching to the third branch in turn.

There are two main reasons the system does not behave identically for different sweep rates. On one hand, slow Joule heating of the crystal causes the overall response to shift in frequency space, as recently demonstrated^10^. Different sweep rates result in different temperatures at a given frequency, thus explaining the amplitude deviations. These deviations may place the system in different basins of attraction for the same frequency, explaining the different moments at which the switch to the third branch occurs. Importantly, despite the frequency at which a transition occurs, afterwards the responses overlap at approximately the same |S_11_| value. Together with the simulated (static) frequency response, this reinforces the interpretation that the reflected signals at different sweep rates indeed represent a transition to the same branch. Furthermore, as the forcing amplitude is increased from 3.75 V to 4.31 V the bandwidth of the third stable branch likewise increases (Fig. 2), as predicted by the theoretical analysis.

On the other hand the theory of dynamic bifurcations plays a role in the observed dependency on sweep rate^36^,^37^. For a finite sweep rate the experimental frequency responses only approximate those given by the static bifurcation diagrams in Fig. 3a–f. The latter assume the response is ‘built up’ by recording a succession of steady-state amplitudes at fixed frequencies. However, in the adiabatic limit of a slowly varying frequency the system trajectory remains within a small neighbourhood (the slow manifold) of the static bifurcation diagram (the critical manifold)^38^. Dynamic bifurcation theory also predicts a delayed bifurcation during passage through a saddle-node point^39^. Therefore the various sweep rates cause the response to lose stability and jump onto the lower energy branch at different points. In reality the exact jump location depends on a combination of Joule heating effects and bifurcation delay. For the purpose of the current analysis it is enough the demonstrate the existence of the third stable branch as predicted by the static bifurcation diagram.

At this point it is clear that any attempt to interpret experimental manifestations of modal coupling—without detailed knowledge of the static bifurcation structure—is prone to ambiguity. Modal interactions elicit a number of dynamic and transient effects that are less pronounced in single degree of freedom systems. As an additional example, before jumping to the lower branch the reflected signal sometimes exhibits a large, narrow dip (Fig. 2b,f and the blue curves in d,e) that has no counterpart in the static frequency response. Therefore, the dips likely correspond to transient interactions between the coupled modes that occur when the system loses stability and begins to transition to the lower energy branch. Future work will study how Joule heating and bifurcation delay complicate the dynamics during loss of stability in modally coupled systems. To separate these effects from phenomena that legitimately reflect the bifurcation structure, such as multistability and quasiperiodic motion due to Hopf points, we have systematically mapped out the structure in Fig. 4. The simulated responses in Fig. 3 indicate the presence of multiple pairs of saddle-node and Hopf points. Numerical continuation of these points in two parameters (forcing amplitude and frequency) explains the evolution of the system from bistability to multistability.

**Figure 5:**
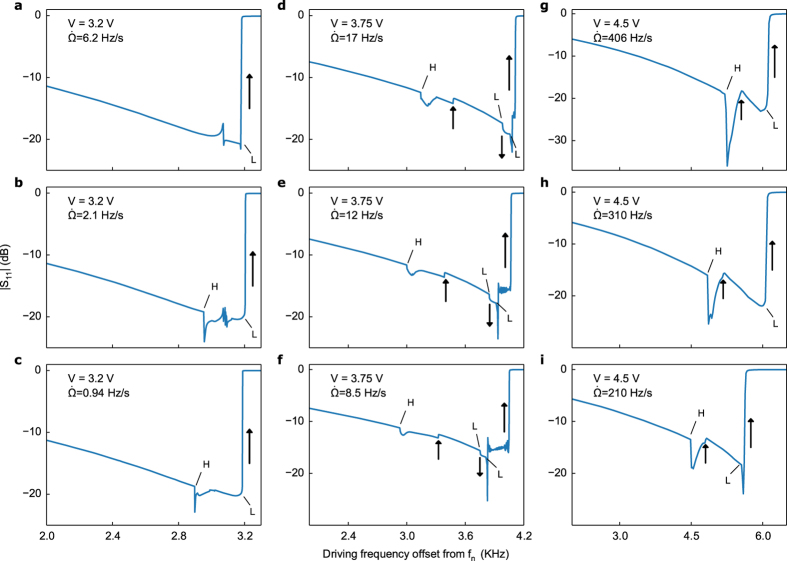
Dynamic effects and quasiperiodic motion. (**a**–**c**) Experimental response at different sweep rates demonstrating Hopf bifurcations and quasiperiodic motion of the resonator. The driving voltage is low enough to preclude the existence of a third stable branch. (**d**–**f**) A variety of multistable and transient effects are observed. (**g**–**i**) Dynamic bifurcation phenomena strongly influence the frequency response at higher sweep rates. Throughout, arrows denote transitions between stable branches, and saddle-node and Hopf bifurcations are denoted by L and H, respectively.

The analysis reveals three nested loci of saddle-node bifurcations (red, blue and purple curves), two of which are connected by a locus of Hopf points (green curve). The red locus of saddle-node points is well-known from standard bistable Duffing behaviour while the others are new predictions of our model that account for multistability. The Hopf curve accounts for some of the more complex behaviour observed in Fig. 5. From about 1–3 KHz offset from *f*_*n*_ in Fig. 4a, the Hopf curve coincides with the blue locus of saddle-node points, which obtain a local minimum and maximum that explain the presence of the isolated loop in Fig. 3a,d. At higher frequencies (>3 KHz offset) the two curves begin to increasingly diverge once the forcing amplitude moves past the local minimum. This coincides with the gradual lengthening of the stable portion of the isolated loop. Indeed, in experiments (Fig. 2) the system begins jumping onto the third branch at around 3.5–4 KHz offset. Once the forcing amplitude exceeds the local maximum, the isolated branch merges with the offshoot from the lower energy branch, as in Fig. 3b,e, reducing the total number of saddle-nodes in a frequency response from six to four. At even higher amplitudes a third locus of saddle-nodes appears (purple curve in Fig. 4; exhibited by the small loop in Fig. 3c,f), although in experiments we did not drive the resonator sufficiently high to observe this.

The ability to observe multistability at feasible drive voltages largely depends on the relationship between *f*_*n*_ and *f*_*m*_ represented by σ_2_. A crucial prediction of our model is that for |σ_2_| large enough the effect of the coupled mode diminishes for realistic forcing amplitudes and the response reverts to standard bistability (for quartz this occurs around |σ_2_| > 2.5 KHz). In fact, linear piezoelectric theory predicts σ_2_ = 27.945 KHz for simple thickness modes (infinite plate approximation), but finite dimensions and electrode geometry cause linear coupling with lateral eigenmodes that can alter this value. Our crystals exhibit σ_2_ ranging between ±17 KHz and we selected crystals with small enough σ_2_ to elicit multistability for realistic drive voltages. For crystals with larger |σ_2_| we did not witness multistability (data not shown). Figure 4a corresponds to our experimental value of σ_2_ while b-d show what theoretically occurs for even smaller |σ_2_|. As |σ_2_| decreases below 1.5 KHz the Hopf and blue saddle-node curves straighten out, destroying their local extrema and thus the isolated branch of the frequency response as well (Fig. 4b–d). In this case the offshoot from the lower energy branch gradually extends to become the third stable branch itself. For σ_2_ < 0 the Hopf curve disappears entirely.

Besides the cusp points (CP) at which saddle-node curves annihilate, a number of other codimension-two bifurcations occur, the most interesting being the Bogdanov-Takens (BT) points that bound the Hopf curve. These indicate the presence of homoclinic bifurcations and often foreshadow more complex behaviour^40^. In Supplementary Section 5 we continue periodic solutions emanating from Hopf points and demonstrate the existence of such homoclinic orbits, along with quasiperiodic motion (periodic solutions for *a*_*n*_ and *a*_*m*_ due to Hopf points imply quasiperiodic motion of the resonator). Such behaviour occurs in regions of the unstable third/isolated branch bounded by Hopf points. While the primary focus of this paper is multistability, in Fig. 5 we experimentally demonstrate some of the more exotic behaviour predicted by our theoretical model. Note that the transient behaviour itself is not directly shown in Figs 3 and 4 but merely implied by the Hopf curve.

For instance, the existence of periodic solutions for *a*_*n*_ and *a*_*m*_ due to Hopf points nicely explains the sudden dips in |S_11_| that precede a transition to the third stable branch in Fig. 5d–i, as well as the rapid oscillations in Fig. 5b distinctive of quasiperiodic motion, or possibly chaos (see also Supplementary Section 5). Since Hopf points exist at amplitudes below which any significant portion of the third branch becomes stable, we should be able to evoke this behaviour before a transition can occur experimentally. Figure 5a–c exhibits this scenario for different sweep rates. At 6.2 Hz/s the system begins to bifurcate to the lower branch, but encounters an attractor of quasiperiodic motion that directs it back to the stable branch before finally bifurcating. By decreasing the sweep rate (Fig. 5b) the system undergoes a Hopf bifurcation, evidenced by the sudden dip in |S_11_|, then loses stability and drifts toward a quasiperiodic (or potentially chaotic) attractor. The reduced sweep rate allows multiple oscillations to be captured in the reflected signal before it returns to the stable branch and bifurcates. At 0.94 Hz/s the system has sufficient time to settle after losing stability and avoids the quasiperiodic attractor. As predicted, these phenomena can occur at lower amplitudes where a transition to the third branch is not yet possible.

Figure 5d–f captures the full range of behaviour, including multistability. After the |S_11_| dip due to the Hopf point, the system returns to the higher energy branch and resides there before transitioning to the third branch. Remarkably, the system returns to the higher energy branch once more before bifurcating. One apparent consequence of this is a more violent transition to the low energy branch, as evinced by the rapid modulation of |S_11_| in Fig. 5e,f after passing the final saddle-node point. Lastly, the different locations of the |S_11_| dip for a given forcing amplitude are due to bifurcation delay, which is predicted for Hopf points just as for saddle-nodes^41^. For high enough sweep rates the system transitions directly from the |S_11_| dip to the third stable branch (Fig. 5g–i).

## Conclusion

In conclusion, nonlinear modal interactions in electroelastic crystals can exhibit incredibly rich, multistable and even quasiperiodic behaviour. The theoretical analysis developed here is potentially useful for a variety of applications that exploit multistability in nonlinear systems. Feedback from modal coupling has already been used to improve frequency stability^28^, and the current work provides a systematic framework to quantitatively model and exploit the effects of modal coupling in general electroelastic crystals. The multistability demonstrated here could potentially be applied in electromechanical memory devices due to the existence of multiple stable states. Furthermore, our experimental results provide a convenient platform to advance fundamental studies of dynamic bifurcations and thermal effects in nonlinear, multiple degree of freedom systems. In future research we aim to more deeply study these interesting transient dynamics.

## Methods

### |S_11_| Measurements

All experimental results were obtained by driving a circular, AT-cut quartz crystal resonator (QCR) around its third thickness-shear harmonic (Laptech Precision, Inc., model XL1191–30.0 M). The QCR was driven with a sinusoidal voltage from an Agilent N5181A signal generator. The signal from the generator was passed through a circulator before reaching the QCR, in order that the reflected signal could be collected by a signal analyzer (Agilent N9000A). Additional details and a diagram of the experimental setup are given in Supplementary Section 6.

### Continuation Analysis

Numerical simulations of the frequency responses and bifurcation diagrams were computed with the AUTO software. The forcing voltage, V, and detuning of the forcing frequency, σ_1_, were used as the continuation parameters. To generate a frequency response for a given forcing amplitude the governing equations were numerically continued from the trivial solution with V = 0 until reaching the desired forcing amplitude. Then, starting from the previous point, the system was continued in σ_1_ to produce the frequency response. To generate the full bifurcation diagram for a given σ_2_ (detuning of the 3:1 internal resonance), the codimension-1 bifurcations identified within a frequency response (saddle-node and Hopf) were continued in both parameters (F and σ_1_). Additional details regarding numerical continuation are given in Supplementary Section 4.

## Additional Information

**How to cite this article**: Kirkendall, C. R. and Kwon, J. W. Multistable internal resonance in electroelastic crystals with nonlinearly coupled modes. *Sci. Rep.* **6**, 22897; doi: 10.1038/srep22897 (2016).

## Supplementary Material

Supplementary Information
