# Supplementary material for: Multistable internal resonance in electroelastic crystals with nonlinearly coupled modes
Source: Sci Rep. 2016 Mar 10;6:22897. doi: 10.1038/srep22897 (PMC4790629; doi:10.1038/srep22897)
Supplement: Supplementary Information [file srep22897-s1.pdf]

Multistable internal resonance in electroelastic crystals with nonlinearly coupled modes

C. R. Kirkendall[1] and J. W. Kwon[1,*]

[1]*Micro-nano Devices and Systems Laboratory, Department of Electrical Engineering, University of Missouri, Columbia, Missouri 65201, USA.* [*]*Correspondence and requests for additional information should be sent to J.K. (email: kwonj@missouri.edu).*

1 Equations of motion for finite deformations

The nonlinear theory of continuous media has been developed at great length by a number of authors[1–3]. Here, we collect some of the main results and sketch a derivation of the equations of motion for an electroelastic crystal. We represent the material body as a Euclidean manifold embedded in $\mathbb{E}^3$. In order to describe finite deformations we must distinguish between the reference (undeformed) and current (deformed) configurations. We refer these to Cartesian systems of rectangular coordinates with bases $\{\mathbf{E_K}\}$ and $\{\mathbf{e_i}\}$, where upper and lowercase indices, respectively, serve to distinguish material and spatial coordinates. The motion of a material point X is given by $x_i = x_i(X_K, t)$ or inversely $X_K = X_K(x_k, t)$. The deformation gradient is then defined by

$$F_{iK} = \frac{\partial x_i}{\partial X_K} = x_{i,K}, \quad F^{-1}_{Ki} = X_{K,i}$$

where a comma denotes partial differentiation. The determinant of the deformation gradient is given by $J = \det x_{i,K}$. The Green and Lagrangian strain tensors are then defined, respectively, as

$$C_{KL} = x_{i,K}x_{i,L}$$
$$E_{KL} = \frac{1}{2}(C_{KL} - \delta_{KL}) = \frac{1}{2}(u_{K,L} + u_{L,K} + u_{M,K}u_{M,L})$$

where $\mathbf{u}$ is the displacement vector that extends from a material point P in the undeformed body to its spatial position in the deformed body, δ_{ij} is the Kronecker delta, and the summation convention over repeated indices is assumed hereafter. The displacement is related

to the reference and current positions by $u_K = \delta_{iK} x_i - X_K$, where δ_{iK} is the shifter that relates components of vectors in the material and spatial coordinates. When the two frames are identical the shifter reduces to the Kronecker delta.

We now collect some key results from a series of papers by Tiersten (see refs 31-33 in the main article) and enforce a notational consistency useful in what follows. The papers (1) derive the equations of electroelasticity based on a macroscopic model of an electronic charge continuum coupled to a lattice continuum and (2) proceed to expand these equations to cubic order in the displacement and electric potential gradients. This lays the groundwork necessary to treat weakly nonlinear dynamics of an electroelastic crystal.

Applying the conservation of mass and linear momentum, along with Maxwell's equations under the quasistatic electric field approximation, we get

$$\rho \dot{v}_j = \tau_{ij,i} + P_i E_{j,i}$$
$$\rho J = \rho_0$$
$$D_{i,i} = 0$$
$$E_i = -\phi_{,i}$$

where ρ is the mass density, ρ_0 is the mass density in the reference configuration, $v_j = \mathrm{d}x_j/\mathrm{d}t$ is the velocity, τ_{ij} is the Cauchy stress tensor, and P_i, E_i, D_i, and ϕ are the the electric polarization, field, displacement, and potential, respectively. The first equation above can be recast in terms of the electrostatic Maxwell stress tensor $T^E_{ij} = D_i E_j - \frac{1}{2}\epsilon_0 E_k E_k \delta_{ij}$ by noting that $T^E_{ij,i} = P_i E_{j,i}$.

$$\rho \dot{v}_j = \tau_{ij,i} + T^E_{ij,i}$$

Now, by defining a thermodynamic state function $\chi = \mathcal{E} - E_i P_i/\rho$, where $\mathcal{E}$ is the stored energy per unit mass, the conservation of energy can be applied to show that

$$\tau_{ij} = \rho x_{i,K} \frac{\partial \chi}{\partial x_{j,K}}$$
$$P_i = -\rho \frac{\partial \chi}{\partial E_i}$$

Furthermore, by applying invariance principles it can be shown that $\chi = \chi(E_{KL}, W_L)$, where $W_L = x_{i,L} E_i = \phi_{,L}$ is the rotationally invariant electrical variable[4,5]. The constitutive

relations then become

$$\tau_{ij} = \rho x_{i,K} x_{j,L} \frac{\partial \chi}{\partial E_{KL}} - P_i E_j$$
$$P_i = -\rho x_{i,L} \frac{\partial \chi}{\partial W_L}$$

To put these equations in a useful form we need to convert them to the reference configuration. Noting that $\tau_{ij} + T_{ij}^E = \tau_{ij}^S + T_{ij}^{ES}$, where $\tau_{ij}^S = \rho x_{i,K} x_{j,L} \partial\chi/\partial E_{KL}$ and $T_{ij}^{ES} = \epsilon_0(E_i E_j - \frac{1}{2} E_k E_k \delta_{ij})$ are symmetric mechanical and Maxwell electrostatic stress tensors, respectively, we arrive at

$$\begin{aligned} K_{Lj,L} &= \rho_0 \dot{v}_j \\ \mathcal{D}_{L,L} &= 0 \end{aligned} \tag{1}$$

where $K_{Lj} = F_{Lj} + M_{Lj}$ is the total first Piola-Kirchhoff stress tensor, whose mechanical and electrical contributions are defined as Piola transforms of τ_{ij}^S and T_{ij}^{ES}.

$$\begin{aligned} F_{Lj} &= J X_{L,i} \tau_{ij}^S = \rho_0 x_{j,K} \frac{\partial \chi}{\partial E_{KL}} \\ M_{Lj} &= J X_{L,i} T_{ij}^{ES} = \epsilon_0 J X_{L,i} (E_i E_j - \frac{1}{2} E_k E_k \delta_{ij}) \end{aligned} \tag{2}$$

and $\mathcal{D}_L$ is the Piola transform of electric displacement.

$$\mathcal{D}_L = J X_{L,i} D_i = J X_{K,i} (\epsilon_0 E_i + P_i) = \epsilon_0 J C_{KL}^{-1} W_L - \rho_0 \frac{\partial \chi}{\partial W_L} \tag{3}$$

Finally, to render the equations tractable we approximate χ using a polynomial expansion about the reference configuration:

$$\begin{aligned} \rho_0 \chi = &\frac{1}{2} c_{ABCD} E_{AB} E_{CD} - e_{ABC} W_A E_{BC} - \frac{1}{2} \chi_{AB} W_A W_B + \frac{1}{6} c_{ABCDEF} E_{AB} E_{CD} E_{EF} \\ &+ \frac{1}{2} k_{ABCDE} W_A E_{BC} E_{DE} - \frac{1}{2} b_{ABCD} W_A W_B E_{CD} - \frac{1}{6} \chi_{ABC} W_A W_B W_C \\ &+ \frac{1}{24} c_{ABCDEFGH} E_{AB} E_{CD} E_{EF} E_{GH} \end{aligned} \tag{4}$$

where c_{ABCD}, e_{ABC}, χ_{AB}, c_{ABCDEF}, k_{ABCDE}, b_{ABCD}, χ_{ABC}, and $c_{ABCDEFGH}$ are the second-order elastic, piezoelectric, second-order electric permeability, third-order elastic, first odd electroelastic, electrostrictive, third-order electric permeability, and fourth order elastic constants, respectively.

2 Weakly nonlinear resonance

We can now expand the equations to cubic order in the displacement gradient and to linear order in the electric potential gradient. Terms involving the electric potential are kept linear because of the relatively small piezoelectric coupling of quartz. First, note that in this case the total Piola-Kirchhoff stress tensor K_{Lj} reduces to F_{Lj}, since M_{Lj} is quadratic in the electric field. We now let the two Cartesian coordinate systems that describe the reference and current configurations coincide. This means that the shifter δ_{jM} is simply the Kronecker delta. Carrying out the expansion to cubic order in $u_{A,B}$ and to linear order in $\phi_{,A}$ gives

$$
\begin{aligned}
K_{Lj}\delta_{jM} = {} & \\
K_{LM} &= c_{LMAB}u_{A,B} + e_{ALM}\phi_{,A} + \frac{1}{2}c_{LMAB}u_{K,A}u_{K,B} + c_{LKAB}u_{M,K}u_{A,B} \\
&\quad + \frac{1}{2}c_{LMABCD}u_{A,B}u_{C,D} + \frac{1}{2}c_{LRAB}u_{M,R}u_{K,A}u_{K,B} + \frac{1}{2}c_{LKABCD}u_{M,K}u_{A,B}u_{C,D} \\
&\quad + \frac{1}{2}c_{LMABCD}u_{A,B}u_{K,C}u_{K,D} + \frac{1}{6}c_{LMABCDEF}u_{A,B}u_{C,D}u_{E,F} \\
D_L &= e_{LAB}u_{A,B} - \epsilon_{LA}\phi_{,A}
\end{aligned}
$$

where $\epsilon_{KL} = \epsilon_0\delta_{KL} + \chi_{KL}$ is the electric permittivity tensor. To simplify these equations we can define effective material constants in terms of fundamental material constants:

$$
\begin{aligned}
K_{LM} &= c_{LMRS}u_{R,S} + e_{KLM}\phi_{,K} + c^{e}_{LMRSKN}u_{R,S}u_{K,N} + c^{e}_{LMRSKNIJ}u_{R,S}u_{K,N}u_{I,J} \\
c^{e}_{LMRSKN} &= \frac{1}{2}(c_{LMRSKN} + c_{LMNS}\delta_{KR}) + c_{LNRS}\delta_{KM} \qquad (5) \\
c^{e}_{LMRSKNIJ} &= \frac{1}{6}c_{LMRSKNIJ} + \frac{1}{2}c_{LMKNSJ}\delta_{RI} + \frac{1}{2}c_{LNSJ}\delta_{MK}\delta_{RI} + \frac{1}{2}c_{LNRSIJ}\delta_{MK}
\end{aligned}
$$

We have developed Mathematica code to expand out a given component of K_{LM}. It is a general code that accommodates tensors of arbitrary rank and automatically symmetrizes them and converts their components to condensed notation. For essentially thickness-shear vibrations in thin plates the boundary conditions on the minor surfaces can be neglected, and in many applications of Y-cut quartz K_{21} and K_{22} are the relevant stress components[6].

In this case only the $u_{1,2}$ and $u_{2,2}$ displacement gradients are retained:

$$K_{21} = c_{66}u_{1,2} + (c_{22} + c_{266})\left(u_{2,2} + \frac{1}{2}u_{2,2}^2\right)u_{1,2} + \left(\frac{1}{2}c_{22} + c_{266} + \frac{1}{6}c_{6666}\right)u_{1,2}^3 + e_{26}\phi_{,2}$$
$$K_{22} = c_{22}u_{2,2} + \frac{3}{2}c_{22}u_{2,2}^2 + \frac{1}{2}(c_{22} + c_{266})(1 + u_{2,2})u_{1,2}^2 + \frac{1}{2}c_{22}u_{2,2}^3$$

Now, for our specific application of thickness-shear resonance u_1 dominates and we consider only K_{21}, which reduces to

$$K_{21} = c_{66}u_{1,2} + \left(\frac{1}{2}c_{22} + c_{266} + \frac{1}{6}c_{6666}\right)u_{1,2}^3 + e_{26}\phi_{,2}$$

Invoking equation (1) and accounting for boundary conditions at the faces of the plate ($X_2 = \pm h$) gives

$$\begin{aligned} c_{66}\hat{u}_{1,22} + e_{26}\hat{\phi}_{,22} + \hat{r}_{66}\dot{\hat{u}}_{1,22} + \hat{\Gamma}[(\hat{u}_{1,2})^3]_{,2} &= \hat{\rho}\ddot{\hat{u}}_1 \\ e_{26}\hat{u}_{1,22} - \epsilon_{22}\hat{\phi}_{,22} &= 0 \\ c_{66}\hat{u}_{1,2} + e_{26}\hat{\phi}_{,2} + \hat{\Gamma}(u_{1,2})^3 &= 0 \quad \text{at } X_2 = \pm h \\ \hat{\phi} \mp \frac{V}{2}\cos\omega\hat{t} &= 0 \quad \text{at } X_2 = \pm h \end{aligned}$$

where

$$\hat{\Gamma} = \frac{1}{2}c_{22} + c_{266} + \frac{1}{6}c_{6666}$$

and V and ω are the amplitude and angular frequency of the voltage applied across the crystal thickness. Hats have been added to denote dimensional quantities and will be removed after nondimensionalization. A linear damping term with coefficient $\hat{r}_{66}$ has been added to account for the small losses of quartz[7]. If we introduce nondimensional quantities defined by

$$y = \frac{X_2}{h}, \quad u_1 = \frac{\hat{u}_1}{h}, \quad \phi = \frac{\hat{\phi}}{\beta}, \quad t = \gamma\hat{t},$$

then the nondimensional equations are

$$\begin{aligned} u_{,22} + \alpha\phi_{,22} + r_{66}\dot{u}_{,22} + \Gamma[(u_{,2})^3]_{,2} &= \rho\ddot{u} \\ u_{,22} - \phi_{,22} &= 0 \\ u_{,2} + \alpha\phi_{,2} + \Gamma(u_{,2})^3 &= 0 \quad \text{at } y = \pm 1 \\ \phi \mp \frac{F}{2}\cos\Omega t &= 0 \quad \text{at } y = \pm 1 \end{aligned} \tag{6}$$

where we have dropped the subscript on u_1 and

$$\alpha = \frac{e_{26}^2}{c_{66}\epsilon_{22}}, \quad r_{66} = \frac{\hat{r}_{66}\gamma}{c_{66}}, \quad \Gamma = \frac{\hat{\Gamma}}{c_{66}}, \quad \gamma = \frac{\pi}{2h}\sqrt{\frac{c_{66}}{\hat{\rho}}}, \quad F = \frac{V}{\beta}, \quad \Omega = \frac{\omega}{\gamma}, \quad \beta = \frac{he_{26}}{\epsilon_{22}}, \quad \rho = \frac{\pi^2}{4}$$

It is worth noting that

$$\frac{\alpha}{1+\alpha} = \frac{e_{26}^2}{\bar{c}_{66}\epsilon_{22}} = k_{26}^2, \quad \text{where } \bar{c}_{66} = c_{66} + \frac{e_{26}^2}{\epsilon_{22}}$$

and k_{26}^2 is the electromechanical coupling coefficient. This completes the derivation of the equations of motion.

3 Perturbation analysis

To obtain equations governing the complex modal amplitudes we employ the method of multiple scales. We seek the solution of equation (6) in the form

$$\begin{aligned} u(y,t;\epsilon) &= \sum_{k=0}^{1} \epsilon^k u_k(y, T_0, T_1) + \cdots \\ \phi(y,t;\epsilon) &= \sum_{k=0}^{1} \epsilon^k \phi_k(y, T_0, T_1) + \cdots \end{aligned} \tag{7}$$

where $\epsilon \ll 1$ is a nondimensional bookkeeping parameter and $T_k = \epsilon^k t$, such that

$$\frac{\partial}{\partial t} = \frac{\partial}{\partial T_0} + \epsilon \frac{\partial}{\partial T_1} + \cdots \equiv D_0 + \epsilon D_1 + \cdots \tag{8}$$

In order that the damping, nonlinearities, and resonances balance each other, we scale r_{66}, Γ, and F as ϵr_{66}, $\epsilon\Gamma$, and ϵF. Substituting equations (7) and (8) into equations (6) and equating coefficients of like powers of ϵ, we obtain

Order ϵ^0:

$$\begin{aligned} u_0'' + \alpha\phi_0'' - \rho D_0^2 u_0 &= 0 \\ u_0'' - \phi_0'' &= 0 \\ u_0' + \alpha\phi_0' &= 0 \quad \text{at } y = \pm 1 \\ \phi_0 &= 0 \quad \text{at } y = \pm 1 \end{aligned} \tag{9}$$

Order ϵ^1:

$$\begin{aligned} u_1'' + \alpha\phi_1'' - \rho D_0^2 u_1 &= -3\Gamma u_0'' \left(u_0'\right)^2 - r_{66} D_0 u_0'' + 2\rho D_0 D_1 u_0 \\ u_1'' - \phi_1'' &= 0 \\ u_1' + \alpha\phi_1' &= -\Gamma (u_0')^3 \quad \text{at } y = \pm 1 \\ \phi_1 &= \pm\frac{F}{2}\cos\Omega T_0 \quad \text{at } y = \pm 1 \end{aligned} \tag{10}$$

where the prime indicates differentiation with respect to y. Equations (9) are the linear homogeneous boundary-value problem associated with equations (6). Its general solution can be expressed as

$$\begin{aligned} u_0(y, T_0, T_1) &= \sum_{l=1}^{\infty} \zeta_l(y) A_l(T_1) e^{i\omega_l T_0} + \text{c.c.} \\ \phi_0(y, T_0, T_1) &= \sum_{l=1}^{\infty} \chi_l(y) A_l(T_1) e^{i\omega_l T_0} + \text{c.c.} \end{aligned}$$

where c.c. means complex conjugate of the preceding terms and the spatial eigenmodes are given by

$$\begin{aligned} \zeta_l(y) &= \frac{\sin \eta_l y}{K_l} \\ \chi_l(y) &= \frac{\sin \eta_l y - y \sin \eta_l}{K_l} \\ \tan \eta_l &= \frac{\eta_l}{k_{26}^2}, \quad K_l = \int_{-1}^{1} \sin^2 \eta_l y \, \mathrm{d}y \end{aligned}$$

However, in the presence of damping, all modes that are not directly excited or indirectly excited through internal resonances decay with time. We account for the primary resonance of the first thickness-shear overtone ($\Omega = \omega_n + \epsilon\sigma_1$) and a three-to-one internal resonance with the fourth overtone ($\omega_m = 3\omega_n + \epsilon\sigma_2$), where σ_1 and σ_2 are $\mathcal{O}(1)$ detuning parameters, such that

$$\begin{aligned} u_0 &= \zeta_n(y) A_n(T_1) e^{i\omega_n T_0} + \zeta_m(y) A_m(T_1) e^{i\omega_m T_0} + \text{c.c.} \\ \phi_0 &= \chi_n(y) A_n(T_1) e^{i\omega_n T_0} + \chi_m(y) A_m(T_1) e^{i\omega_m T_0} + \text{c.c.} \end{aligned} \tag{11}$$

Substituting equations (11) into equations (10) gives

$$
\begin{aligned}
u_1'' + \alpha\phi_1'' - \rho D_0^2 u_1 = {} & e^{i\omega_n T_0}[2i\omega_n\rho\zeta_n D_1 A_n - i\omega_n r_{66}\zeta_n'' A_n \\
& - 6\Gamma(\zeta_n''(\zeta_m')^2 + 2\zeta_m'\zeta_m''\zeta_n')A_m A_n \bar{A}_m - 9\Gamma\zeta_n''(\zeta_n')^2 A_n^2\bar{A}_n] \\
& + e^{i\omega_m T_0}[2i\omega_m\rho\zeta_m D_1 A_m - i\omega_m r_{66}\zeta_m'' A_m \\
& - 6\Gamma(\zeta_m''(\zeta_n')^2 + 2\zeta_m'\zeta_n'\zeta_n'')A_m A_n \bar{A}_n - 9\Gamma\zeta_m''(\zeta_m')^2 A_m^2\bar{A}_m] \\
& - 3\Gamma e^{i(\omega_m - 2\omega_n)T_0}[\zeta_m''(\zeta_n')^2 - 2\zeta_m'\zeta_n'\zeta_n'']A_m\bar{A}_n^2 \\
& - 3\Gamma e^{3i\omega_n T_0}\zeta_n''(\zeta_n')^2 A_n^3 + \text{c.c} + \text{NST}
\end{aligned}
\tag{12}
$$

$$
u_1'' - \phi_1'' = 0 \tag{13}
$$

$$
\begin{aligned}
u_1' + \alpha\phi_1' = {} & -3\Gamma e^{i\omega_n T_0}[2\zeta_n'(\zeta_m')^2 A_m A_n\bar{A}_m + (\zeta_n')^3 A_n^2\bar{A}_n] \\
& - 3\Gamma e^{i\omega_m T_0}[2\zeta_m'(\zeta_n')^2 A_m A_n\bar{A}_n + (\zeta_m')^3 A_m^2\bar{A}_m] \\
& - 3\Gamma e^{i(\omega_m - 2\omega_n)T_0}\zeta_m'(\zeta_n')^2 A_m\bar{A}_n^2 \\
& - \Gamma e^{3i\omega_n T_0}(\zeta_n')^3 A_n^3 + \text{c.c} + \text{NST} \quad \text{at } y = \pm 1
\end{aligned}
\tag{14}
$$

$$
\phi_1 = \pm\frac{F}{4}\left(e^{i\Omega T_0} + e^{-i\Omega T_0}\right) \quad \text{at } y = \pm 1 \tag{15}
$$

where NST stands for terms that do not produce secular terms. Since the homogeneous part of equations (12)-(15) has a nontrivial solution, the nonhomogeneous system has a solution only if solvability conditions are met[8,9]. Imposing the solvability conditions gives equations governing the complex modal amplitudes A_n and A_m:

$$
\begin{aligned}
& 2i(D_1 A_n + \mu_n A_n) + 8\gamma_{nn}A_n^2\bar{A}_n + 8\gamma_{nm}A_m A_n\bar{A}_m + 8\gamma_{n0}A_m\bar{A}_n^2 e^{-iT_1\sigma_2} = f e^{iT_1\sigma_1} \\
& 2i(D_1 A_m + \mu_m A_m) + 8\gamma_{mm}A_m^2\bar{A}_m + 8\gamma_{mn}A_m A_n\bar{A}_n + 8\gamma_{m0}A_n^3 e^{-iT_1\sigma_2} = 0
\end{aligned}
\tag{16}
$$

where

$$
\begin{aligned}
8\rho\omega_n\gamma_{nn} &= -3\Gamma\left(\xi_n(-1)\zeta_n'(-1)^3 - \xi_n(1)\zeta_n'(1)^3 + 3\int_{-1}^{1}\xi_n(y)\zeta_n'(y)^2\zeta_n''(y)\,\mathrm{d}y\right)\\
4\rho\omega_n\gamma_{nm} &= -3\Gamma\Bigg(\xi_n(-1)\zeta_n'(-1)\zeta_m'(-1)^2 - \xi_n(1)\zeta_n'(1)\zeta_m'(1)^2\\
&\qquad + 2\int_{-1}^{1}\xi_n(y)\zeta_m'(y)\zeta_n'(y)\zeta_m''(y)\,\mathrm{d}y + \int_{-1}^{1}\xi_n(y)\zeta_m'(y)^2\zeta_n''(y)\,\mathrm{d}y\Bigg)\\
8\rho\omega_n\gamma_{n0} &= -3\Gamma\Bigg(\xi_n(-1)\zeta_m'(-1)\zeta_n'(-1)^2 - \xi_n(1)\zeta_m'(1)\zeta_n'(1)^2\\
&\qquad + 2\int_{-1}^{1}\xi_n(y)\zeta_m'(y)\zeta_n'(y)\zeta_n''(y)\,\mathrm{d}y + \int_{-1}^{1}\xi_n(y)\zeta_n'(y)^2\zeta_m''(y)\,\mathrm{d}y\Bigg)\\
8\rho\omega_m\gamma_{mm} &= -3\Gamma\left(\xi_m(-1)\zeta_m'(-1)^3 - \xi_m(1)\zeta_m'(1)^3 + 3\int_{-1}^{1}\xi_m(y)\zeta_m'(y)^2\zeta_m''(y)\,\mathrm{d}y\right)\\
4\rho\omega_m\gamma_{mn} &= -3\Gamma\Bigg(\xi_m(-1)\zeta_m'(-1)\zeta_n'(-1)^2 - \xi_m(1)\zeta_m'(1)\zeta_n'(1)^2\\
&\qquad + 2\int_{-1}^{1}\xi_m(y)\zeta_m'(y)\zeta_n'(y)\zeta_n''(y)\,\mathrm{d}y + \int_{-1}^{1}\xi_m(y)\zeta_n'(y)^2\zeta_m''(y)\,\mathrm{d}y\Bigg)\\
8\rho\omega_m\gamma_{m0} &= -\Gamma\left(\xi_m(-1)\zeta_n'(-1)^3 - \xi_m(1)\zeta_n'(1)^3 + 3\int_{-1}^{1}\xi_m(y)\zeta_n'(y)^2\zeta_n''(y)\,\mathrm{d}y\right)\\
f &= \frac{F}{4\rho\omega_n}\left(\psi_n'(1) + \psi_n'(-1) - \alpha(\xi_n'(1) + \xi_n'(-1))\right)\\
\mu_n &= \frac{r_{66}\eta_n^2}{2\rho}, \quad \mu_m = \frac{r_{66}\eta_m^2}{2\rho}
\end{aligned}
$$

where $\xi_l(y)$ and $\psi_l(y)$ ($l = n, m$) are spatial eigensolutions of the adjoint homogeneous problem associated with equations (12)-(15), and are given by

$$
\begin{aligned}
\xi_l(y) &= \frac{\sin\eta_l y}{K_l}\\
\psi_l(y) &= \frac{\alpha(\sin\eta_l y - y\sin\eta_l)}{K_l}
\end{aligned}
$$

It should be noted that equivalent equations arise if the nonhomogeneous boundary terms in equations (14) and (15) are first shifted onto equations (12) and (13). In this case the solvability conditions require that the right-hand sides of equations (12) and (13) be orthogonal to every solution of the adjoint homogeneous problem. However, this approach obscures the origin of contributions to the coupling coefficients that arise from the boundary terms.

For AT-cut quartz we have that

$$\gamma_{nn} = 881.412268, \quad \gamma_{nm} = 10580.640673, \quad \gamma_{n0} = 880.793874$$

$$\gamma_{mm} = 23798.125533 \quad \gamma_{mn} = 3525.785362, \quad \gamma_{m0} = 97.835605$$

$$f = -2.664704 \times 10^{-9}V, \quad \omega_n = 3.010644, \quad \omega_m = 9.034738$$

$$\eta_n = 4.710743, \quad \eta_m = 14.136618, \quad \Gamma = 47.225095$$

Note that $\omega_n\gamma_{nm} = \omega_m\gamma_{mn}$, which is necessary if equations (16) are to be derivable from a Lagrangian.

We convert equations (16) to a set of real-valued autonomous ODEs via the transformations

$$A_n \to \frac{1}{2}(p_n - iq_n)e^{iT_1\sigma_1}, \quad A_m \to \frac{1}{2}(p_m - iq_m)e^{iT_1(3\sigma_1-\sigma_2)}$$

and set $\epsilon = 1$, which gives

$$\begin{aligned}
p_n'(t) &= \gamma_{nn}q_n\left(p_n^2+q_n^2\right)+\gamma_{nm}q_n\left(p_m^2+q_m^2\right)+\gamma_{n0}\left(p_n\left(q_mp_n-2p_mq_n\right)-q_mq_n^2\right)-\mu_np_n-\nu_1q_n\\
q_n'(t) &= f-\gamma_{nn}p_n\left(p_n^2+q_n^2\right)-\gamma_{nm}p_n\left(p_m^2+q_m^2\right)+\gamma_{n0}\left(p_mq_n^2-p_n\left(p_mp_n+2q_mq_n\right)\right)-\mu_nq_n+\nu_1p_n\\
p_m'(t) &= \gamma_{mm}q_m\left(p_m^2+q_m^2\right)+\gamma_{mn}q_m\left(p_n^2+q_n^2\right)+\gamma_{m0}q_n\left(3p_n^2-q_n^2\right)-\mu_mp_m-\nu_2q_m\\
q_m'(t) &= -\gamma_{mm}p_m\left(p_m^2+q_m^2\right)-p_m\gamma_{mn}\left(p_n^2+q_n^2\right)+\gamma_{m0}p_n\left(3q_n^2-p_n^2\right)-\mu_mq_m+\nu_2p_m
\end{aligned} \tag{17}$$

where $\nu_1 = \sigma_1$ and $\nu_2 = 3\sigma_1 - \sigma_2$. The modal amplitudes are then given by $a_l = \sqrt{p_l^2 + q_l^2}$

Finally, before carrying out numerical continuation we redimensionalize these equations as follows:

$$p_l \to p_l\left(\frac{\mu_n}{\gamma_{nn}}\right)^{1/3}, \quad q_l \to q_l\left(\frac{\mu_n}{\gamma_{nn}}\right)^{1/3}, \quad t \to t\left(\frac{1}{\gamma_{nn}\mu_n^2}\right)^{1/3} \tag{18}$$

4 Numerical continuation

All numerical continuation results were obtained using the AUTO software. Plots of the primary and coupled mode amplitudes (a_n and a_m) were obtained by simulating equations (17), after redimensionalization via equations (18). The amplitudes were extracted as $a_l =$

$\sqrt{p_l^2 + q_l^2}$, $l = n, m$. In plots containing both equilibria and periodic solutions (e.g. Fig. S1 below), the L^2 norm is used. From the AUTO-07p manual this is calculated as

$$L^2 \text{ norm} = \sqrt{\int_0^1 \sum_{k=1}^{NDIM} U_k(t)^2 \mathrm{d}t}$$

where the $U_k(t)$ are the p_n, p_m, q_n, and q_m in equation (17). Note that the integration limits of [0,1] occur since AUTO transforms the independent variable to range from 0 to 1 for periodic solutions.

As explained in the main article, the finite dimensions and electrode geometry of experimental crystals cause the thickness-shear resonances to couple with lateral eigenmodes. This does not appear to cause qualitative changes in the bifurcation structure for AT-cut quartz, as shown in analyses using approximate Mindlin plate equations that account for lateral resonances[10–13]. However, it does cause multistable behavior to occur at lower driving voltages than predicted theoretically. Since the theoretical results also predict multistable behavior at progressively lower voltages as σ_2 decreases (see Fig. 4 in the main article), we used an effective value for σ_2 of 1.8 KHz to compute our theoretical frequency responses, instead of the experimentally determined value of $\sigma_2 = 2.25$ KHz. As a result, the theoretical drive voltages more closely match those at which multistability occurs in experiment. Accounting for the effect of Joule heating would also slightly modify the effective spring constants of each mode, and in turn the effective value of σ_2. Even without these extra considerations the agreement is still quite close, though in future work we intend to incorporate the effects of Joule heating and coupling with lateral eigenmodes.

5 Homoclinic orbits and quasiperiodic motion

One advantage of numerical continuation methods is the ability to continue periodic solutions emanating from Hopf bifurcations of equilibria. Figure S1 demonstrates this for the isolated loop of a representative frequency response. The periodic branches undergo a series of period doubling bifurcations and rapidly increase in amplitude. This accounts for the $|S_{11}|$

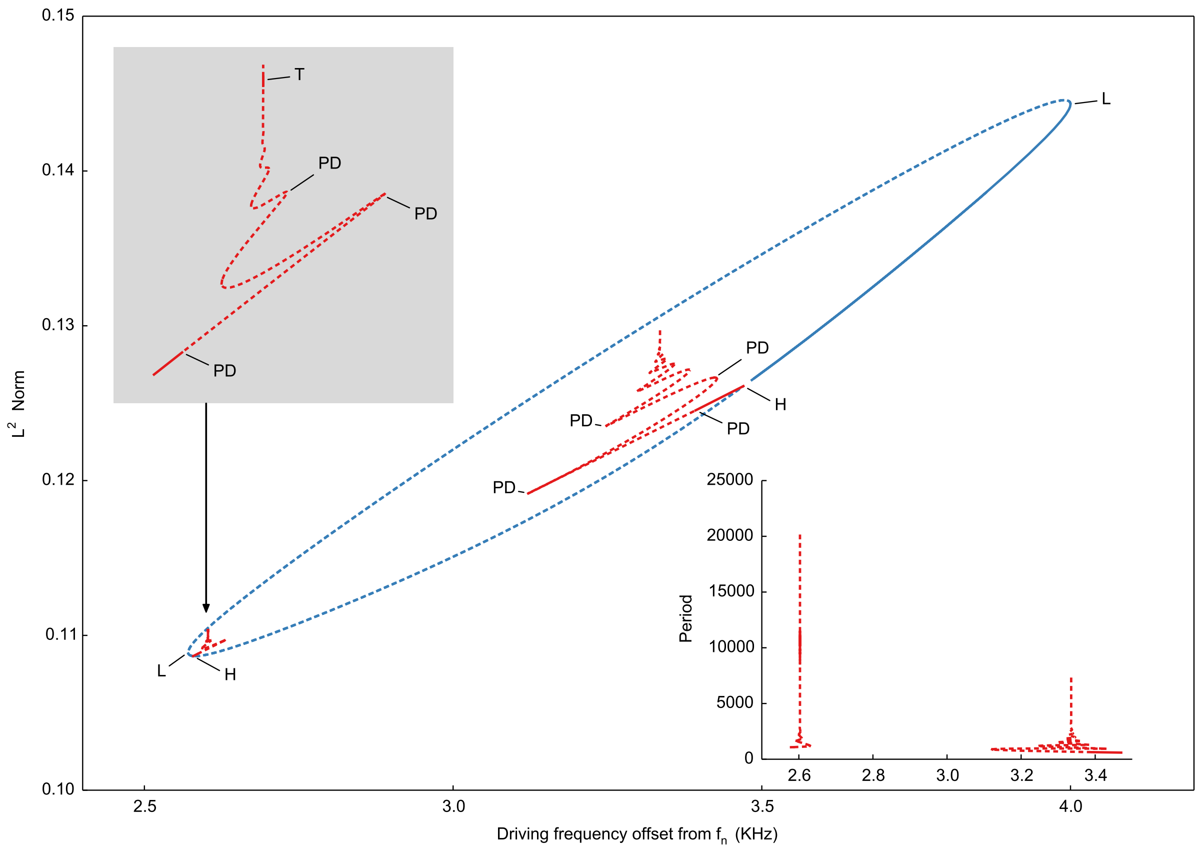

Figure S1: Continuation of periodic solutions (red curves) emanating from Hopf bifurcations of the isolated loop (blue curve). Labels PD, T, H, and L denote period doubling, torus, Hopf, and saddle-node bifurcations, respectively. Solid (dashed) lines denote stable (unstable) solutions. The inset plots the period of the periodic branches against the driving frequency offset and the grey box is a blown up version of the first periodic branch.

dips in the experimental response (see Fig. 5 in the main article). As the amplitude of the periodic branches increases, their period likewise increases, effectively tending to infinity. Such behavior indicates the presence of homoclinic orbits. Furthermore, the continuation detects a torus bifurcation, and both suggest the existence of complex dynamics in the region between the two Hopf points. While these results anticipate the more complex transient behaviour witnessed in Fig. 5, it should be emphasized that the specific nature of the transient dynamics cannot be directly read from Fig. S1. Proving the existence of periodic orbits only tells us *where* more complex transient behavior might occur and not exactly how it will manifest. Specifically, we know such behaviour should occur in the region bounded by the two Hopf points in Fig. S1. Indeed, the experimental results corroborate these predictions as noted in the main article. Lastly, the main purpose of the model is to predict the steady state solutions and whether multistability is possible. The further prediction of experimentally observed transient behaviour is an added bonus, but future work is needed to unravel the specifics.

6 Experimental Setup

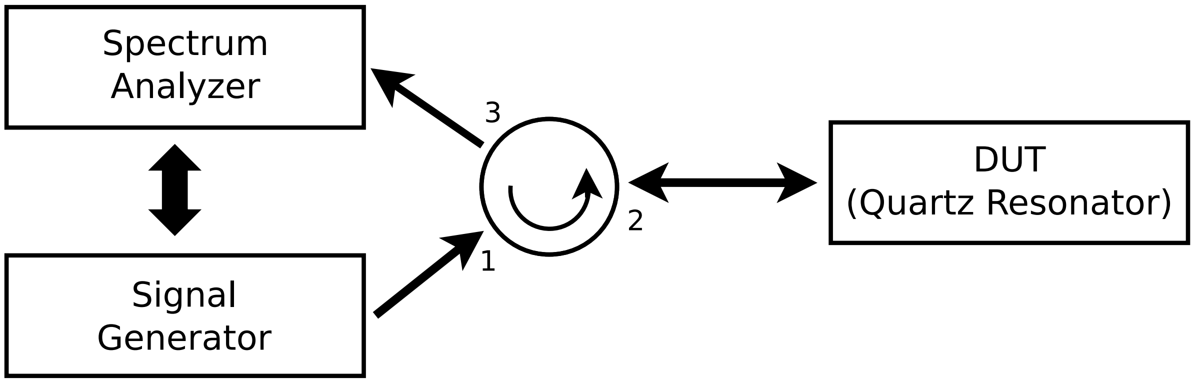

Figure S2: Diagram of experimental setup. The spectrum analyzer uses built-in software to control the signal generator and collect the frequency response of the device. An RF circulator routes the signal from the source to the quartz resonator and the reflected signal is directed to the spectrum analyzer.

A schematic of the test setup is given in Fig. S2. To measure the frequency response of the quartz resonator the signal generator is controlled by the spectrum analyzer. A single sinusoidal signal is swept around 30 MHz at various drive voltages to produce the plots in Figs. 1a, 2 and 5 of the main article. For large enough driving amplitudes two modes are excited: the directly forced thickness-shear mode near 30 MHz and the internally resonant mode near 90 MHz (i.e. a 1:3 internal resonance between the third and ninth harmonics).

References

1. Eringen, A. C. *Mechanics of Continua* (Krieger Publishing Co., 1980).

2. Maugin, G. A. *Nonlinear Electromechanical Couplings* (John Wiley & Sons Incorporated, 1992).

3. Liu, I.-S. *Continuum Mechanics* (Springer Science & Business Media, 2002).

4. Toupin, R. A. The elastic dielectric. *Journal of Rational Mechanics and Analysis* **5**, 849–915 (1956).

5. Eringen, A. C. On the foundations of electroelastostatics. *Int. J. Eng. Sci.* **1**, 127–153 (1963).

6. Tiersten, H. Analysis of nonlinear resonance in thickness-shear and trapped-energy resonators. *J. Acoust. Soc. Am.* **59**, 866–878 (1976).

7. Gagnepain, J. & Besson, R. *Nonlinear effects in piezoelectric quartz crystals*, vol. 11, 245–288 (Academic Press New York, 1975).

8. Nayfeh, A. H. *Introduction to Perturbation Techniques* (John Wiley & Sons, 2011).

9. Nayfeh, A. & Bouguerra, H. Non-linear response of a fluid valve. *Int. J. Nonlinear Mech.* **25**, 433–449 (1990).

10. Wu, R., Wang, J., Du, J., Huang, D. & Hu, Y. The non-linear thickness-shear vibrations of quartz crystal plates under an electric field. *Int. J. Nonlinear Mech.* **61**, 32–38 (2014).

11. Wu, R. *et al.* An analysis of nonlinear vibrations of coupled thickness-shear and flexural modes of quartz crystal plates with the homotopy analysis method. *IEEE Trans. Ultrason. Ferroelectr. Freq. Control* **59**, 30–39 (2012).

12. Yang, Z., Hu, Y., Wang, J. & Yang, J. Nonlinear coupling between thickness-shear and thickness-stretch modes in a rotated y-cut quartz resonator. *IEEE Trans. Ultrason. Ferroelectr. Freq. Control* **56**, 220–224 (2009).

13. Yang, J. & Shen, X. Coupling to extension in a thickness-shear resonator due to relatively large thickness-shear deformation. *IEEE Trans. Ultrason. Ferroelectr. Freq. Control* **55**, 726–729 (2008).
